# Altered white matter microarchitecture in amyotrophic lateral sclerosis: A voxel-based meta-analysis of diffusion tensor imaging

**DOI:** 10.1016/j.nicl.2018.04.005

**Published:** 2018-04-04

**Authors:** Feifei Zhang, Guangxiang Chen, Manxi He, Jing Dai, Huifang Shang, Qiyong Gong, Zhiyun Jia

**Affiliations:** a Huaxi MR Research Center (HMRRC), Department of Radiology, West China Hospital of Sichuan University, Chengdu 610041, China; b Department of Radiology, The Affiliated Hospital of Southwest Medical University, Luzhou 646000, China; c Department of Nuclear Medicine, West China Hospital, Sichuan University, Chengdu 610041, China; d Department of Psychoradiology, Chengdu Mental Health Center, Chengdu 610031, China; e Department of Neurology, West China Hospital, Sichuan University, Chengdu 610041, China

**Keywords:** Diffusion tensor imaging, Amyotrophic lateral sclerosis, MRI, Psychoradiology

## Abstract

**Background:**

The results of recent diffusion tensor imaging (DTI) studies on amyotrophic lateral sclerosis (ALS) are inconclusive and controversial. We performed a voxel-based meta-analysis to identify a statistical consensus among published DTI studies of altered white matter (WM) microarchitecture in ALS.

**Methods:**

A systematic search was conducted for relevant studies that used voxel-wise analyses of WM microarchitecture in patients with ALS. Anisotropic effect size-signed differential mapping (AES-SDM) was applied to analyze fractional anisotropy (FA) differences between ALS patients and healthy controls. Meta-regression analysis was used to explore the effects of clinical characteristics on WM integrity in patients with ALS.

**Results:**

A total of 14 studies with 16 datasets that included 396 patients and 360 healthy controls were identified. The pooled meta-analysis revealed that patients with ALS exhibited significant FA reductions in two clusters relative to healthy controls. The largest cluster exhibited a peak coordinate in the left corona radiata, extending to the body and splenium of the corpus callosum, left superior longitudinal fasciculus, posterior limb of the internal capsule, right corona radiata, and bilateral cingulate gyrus. The other cluster exhibited decreased FA in the right corticospinal tract that extended to the right cerebral peduncle. The Amyotrophic Lateral Sclerosis Functional Rating Scale-Revised (ALSFRS-R) score was positively correlated with the FA reduction in the left corona radiata. Mean age and illness duration were not linearly correlated with the FA reductions.

**Conclusions:**

This study provides a thorough profile of WM microarchitecture alterations in patients with ALS and further evidence that the neuronal degeneration is not limited to the corticospinal tract but also includes extra-motor areas, which supports the view that ALS is a multisystem degenerative disorder that involves the white matter.

## Highlights

• To identify statistical consensus between published DTI studies in ALS
• Two consistent clusters of FA reductions in patients with ALS.
• ALS is a multisystem degenerative disorder involving the WM.

## 1 Introduction

Amyotrophic lateral sclerosis (ALS) is a neurodegenerative, age-related and predominantly male disease that may result in the progressive loss of bulbar and limb function. **Patients suffer from motor function deteriorated and finally develop fatal respiratory failure (****Schmidt et al., 2014****)**. ALS is a disease with an insidious onset, and there is a long period before typical symptoms begin appearing. The overall lifetime prevalence rate is 1:400, and most patients die within 3–5 years of symptom onset (O et al., 2011). The diagnosis is based on upper and lower motor neuron clinical signs (O et al., 2011). **With the development of imaging technology, magnetic resonance imaging (MRI) is now the leading technique in the search for biomarkers (****Turner et al.,** 2011**) and currently assists with the diagnosis of ALS substantially**.

In recent years, psychoradiology (Lui et al., 2016) which studies brain diseases through a variety of non-invasive imaging techniques is emerging. Diffusion tensor imaging (DTI) is a mature and sensitive instrument for detecting WM microarchitecture track alterations. DTI measurements depend on the fractional anisotropy (FA), which is greater in major WM tracks, lower in gray matter, and approaches 0 in cerebrospinal fluid (Smith et al., 2006). A previous study (Thivard et al., 2007) demonstrated that FA alterations result from a loss of fiber integrity caused by axonal degeneration as indicated by the lack of volume loss. Diffusion MRI is a promising and non-invasive method to detect the degree of fiber damage in various disease processes that affect WM by measuring FA. There is a large body of evidence highlighting FA changes in ALS that have used voxel-based analyses (VBA) or tract-based spatial statistics (TBSS) methods. However, the results are inconsistent and controversial. According to previous studies, a significant decrease in FA has been observed in patients with ALS in WM regions that include the bilateral corticospinal tract (CST) (Crespi et al., 2014; Kassubek et al., 2014; Senda et al., 2011), corpus callosum (CC) fibers (Agosta et al., 2007; Keil et al., 2012), thalamus (Sach et al., 2004; Thivard et al., 2007), right association fibers (Zhang et al., 2014), right frontal subgyral area and left frontal precentral area (Abe et al., 2004). One study found an FA reduction in the bilateral posterior portion of the corona radiata and the left cerebral peduncle (Zhang et al., 2007). However, another study found FA reductions in the right cerebral peduncle and left posterior limb of the internal capsule (Zhang et al., 2014). An intriguing study (Trojsi et al., 2015) divided ALS patients into 3 stages according to clinical diagnoses and found decreased FA in the body of the CC and the left CST in clinical stage 2A patients, in the left cerebellar hemisphere, brainstem precerebellar nuclei and premotor cortex in clinical stage 2B patients, and in the rostral part of the CSTs, body of the CC, thalamic radiations, bilateral superior and inferior longitudinal and fronto-occipital fasciculi, right uncinate fasciculus and midbrain in clinical stage 3 patients. However, another study found no significant differences in the central part of the CC between patients with ALS and healthy controls (Agosta et al., 2010). Thus, conducting a meta-analysis to identify the most prominent and consistent statistical results from DTI studies of the WM integrity of patients with ALS is necessary.

A previous meta-analysis found FA reductions in the bilateral frontal WM and the posterior limb of the bilateral internal capsule (Li et al., 2012). However, only 8 published DTI studies were included in this meta-analysis, and other confounding factors, such as possible methodological differences between the included studies, were not considered. Additionally, this study did not use meta-regression to investigate the potential moderating effects of clinical characteristics on regional WM abnormalities. Recently, there have been many original studies on this issue. To overcome these limitations and increase our understanding of this neurobiological disorder, we intend to identify and integrate more recent DTI studies for a more comprehensive and rigorous meta-analysis, which will be the most up-to-date meta-analysis available.

Our purposes in conducting this meta-analysis are as follows: first, to quantitatively summarize the 14 most relevant DTI studies (16 datasets met the inclusion criteria) concerning FA abnormalities in patients with ALS using a newly developed meta-analysis technique, anisotropic effect size-signed differential mapping (AES-SDM), which has the potential to quantify the reproducibility of neuroimaging findings and produce insights that are difficult to obtain from individual studies; second, to perform subgroup meta-analyses that include only studies with methodological homogeneity to avoid the potentially confounding effects of different methodologies; third, to use a meta-regression method to assess the potential effects of mean age, age at onset, duration of disease, and ALSFRS-R score on the reported WM abnormalities.

## 2 Materials and methods

### 2.1 Literature search strategy

We searched PubMed, EMBASE, Web of Science, Cochrane Library and Science Direct to find relevant literature published articles between January 1994 and November 2016 and “in press” articles. All follow a systematic and extensive retrieval strategy. The search keywords were (“amyotrophic lateral sclerosis” or “ALS”) and (“diffusion tensor” or “DTI” or “diffusion magnetic resonance imaging”). Additionally, the reference lists of both eligible articles and review articles were manually screened to avoid omitting.

### 2.2 Study selection and data extraction

All studies found in our search were assessed. The studies included in the meta-analysis met all of the following inclusion criteria: (i) original articles published in peer-reviewed English-language journals; (ii) studies comparing the FA values of WM areas between patients with ALS and healthy controls; and (iii) studies that detected FA alterations in whole-brain analyses and reported the results in Talairach or Montreal Neurological Institute coordinates. The corresponding authors of studies that met all of the above inclusion criteria but lacked global brain coordinates were contacted to obtain additional information.

The exclusion criteria for this meta-analysis were as follows: (i) studies that were case reports or reviews; (ii) studies involving familial ALS patients; (iii) studies that lacked an health control group; and (iv) studies using overlapping research samples from different publications (in such cases, the data from the study with the largest sample were included in the meta-analysis). Additionally, our meta-analysis also defers to the guidelines of Meta-analysis of Observational Studies in Epidemiology (MOOSE) (Stroup et al., 2000).

Two authors extracted from the included studies demographic characteristics (sample size, age, and gender), clinical data (age at onset, illness duration, symptom severity), data processing method, statistical thresholds. **The three-dimensional coordinates in each study were also extracted for the meta-analysis according to the AES-SDM methods (****Radua et al., 2014b****). Its main feature is the possibility of combining peak coordinates and statistical parameter map, and use of statistical data has been established and the differences between accounting research (****Radua et al., 2012b****)**. The authors did this independently and based on the AES-SDM method (Radua et al., 2014b). Discussions were performed when there are disagreements.

### 2.3 Voxel-wise meta-analysis

We performed a voxel analysis to identify FA differences between ALS patients and healthy controls, and AES-SDM software was used according to standard procedures. To examine the reproducibility, we performed systematic whole-brain voxel-based jack-knife sensitivity analysis. This method consists of iteratively repeating the pooled meta-analysis while discarding a different data set in each iteration to determine whether a discovery was replicable (Radua et al., 2014a). We also performed several subgroup meta-analyses to analyze methodological differences between these studies. This analysis included only the studies with methodological homogeneity, which was repeated for those studies that used VBA or TBSS approaches, for those that used statistical parametric mapping (SPM) and the functional MRI of the brain (FMRIB) software library (FSL) or tensor imaging and fiber tracking (TIFT) software, and for those with corrected or uncorrected thresholds.

All analytical processes above were based on the AES-SDM tutorial (http://sdmproject.com/software/Tutorial.pdf) and previous literature (Radua et al., 2014a). The parameters of AES-SDM were as follows: anisotropy = 1.0; isotropic full-width at half-maximum (FWHM) = 20 mm; peak height threshold = 1; voxel *p* = 0.005; and cluster extent = 10 voxels with 10 repetitions of standard randomization tests. Furthermore, to convert the AES-SDM results into imaging, we used MRIcron software (http://www.cabiatl.com/mricro/mricron/), and the results were overlaid on a high-resolution brain image template created by the International Consortium for Brain Mapping. The WM bands were plotted with DTI query software (http://graphics.stanford.edu/projects/dti/) by displaying the brain regions with significant FA differences and by means of a map of the human WM anatomy (Wakana et al., 2004). We used the pre-computed paths from DTI sample data from a normal 35-year-old male subject that were provided by the DTI query software.

### 2.4 Meta-regression analysis

Considering the potential influences of mean age, age at onset, duration of disease, and ALSFRS-R score on WM abnormalities, we performed a meta-regression analysis. The primary output for each of the variables above was shown on the regression slope plot. To minimize the risk of false positive or false negative findings, we chose a low probability threshold of 0.0005 according to previous meta-analyses and the recommendations of the AES-SDM authors (Radua and Mataix-Cols, 2009). Only extreme slopes and regressors were reported in our meta-analysis, and others that did not exist in the main analysis were ignored. Additionally, to avoid discarding data that were driven by insufficient research, the regression maps were visually inspected (Radua et al., 2012a).

## 3 Results

### 3.1 Included studies and sample characteristics

**Fig. 1:**
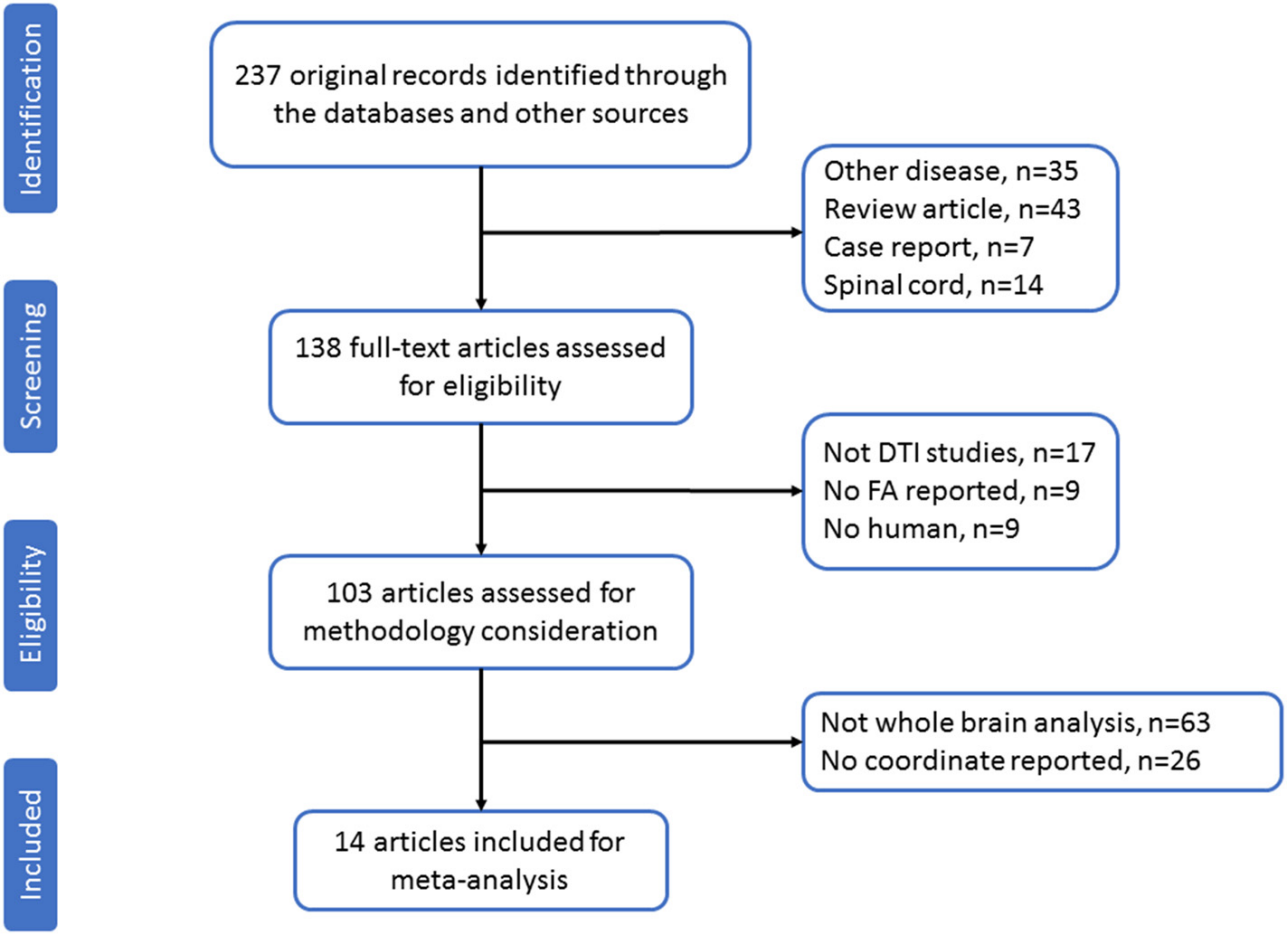
Flow diagram for the identification and exclusion of studies in patients with ALS. ALS = amyotrophic lateral sclerosis.

We searched based on the above strategy and identified a total of 234 studies. According to our inclusion and exclusion criteria, only 14 of these studies with 16 datasets met the inclusion criteria. These studies reported the FA abnormalities of the WM in 396 patients with ALS (235 males and 161 females; mean age 59.2 ± 9.7 years) compared with 360 healthy controls (210 males and 150 females; mean age 63.29 ± 10.0 years). In one study (Trojsi et al., 2015), the ALS patients were divided into 3 groups according to clinical stage, and these groups were compared with the same healthy controls. We, therefore, treated this study as three unique and independent datasets in our meta-analysis. A flow diagram of the identification and the attributes of the studies is presented in Fig. 1, and Table 1 summarizes the characteristics of all studies included in the meta-analysis.

**Table 1:** Demographic and clinical characteristics of the participants in 14 studies (16 datasets) on amyotrophic lateral sclerosis included in the meta-analysis.

Study	Subjects, n (females, n)		Age, years		Onset		Age at onset years, mean	Illness duration, months	ALSFRS-R	Software	Methods	Statistical threshold
	Patients	Controls	Patients	Controls	Bulbar	Limb						
Abe et al., 2004 [19]	7(4)	11(6)	57.3 ± 6.2	57.1 ± 4.5	NA	NA	NA	19.6 ± 12.8	NA	SPM99	VBA	*p* < 0.05 (corrected)
Sach et al., 2004 [20]	15(5)	12(NA)	52.2 ± 11.8	52.8 ± 10.9	6	9	NA	11.9 ± 5.6	NA	SPM99	VBA	*p* < 0.01 (uncorrected)
Agosta et al., 2007 [21]	25(11)	18(7)	54.1 ± NA	52.2 ± NA	6	19	NA	39.0 ± NA	29.0 ± 4.3	SPM2	VBA	*p* < 0.001 (uncorrected)
Sage et al., 2007 [22]	28(14)	26(15)	58.9 ± 11.8	53.7 ± 11.8	6	21	NA	14.6 ± 8.2	39.7 ± 6.3	SPM2	VBA	*p* < 0.05 (FWE)
Thivard et al., 2007 [23]	15(6)	25(11)	51.8 ± 8.7	44.9 ± 12.4	4	11	NA	30.9 ± 15.9	30.0 ± 6.0	SPM2	VBA	*p* < 0.05 (FDR)
Zhang et al., 2007 [23]	8(2)	8(2)	60.0 ± 11.0	46.0 ± 6.0	NA	NA	NA	NA	NA	SPM2	VBA	*p* < 0.05 (FWE)
Senda et al., 2011[31]	17(9)	17(9)	61.3 ± 7.8	60.7 ± 7.2	5	12	NA	38.4 ± 19.2	36.7 ± 6.1	SPM5	VBA	*p* < 0.001 (uncorrected)
Keil et al., 2012 [32]	24(11)	24(10)	62.4 ± 10.5	61.6 ± 9.2	9	15	NA	25.6 ± 27.8	36.3 ± 9.0	SPM2	VBA	*p* < 0.05 (FWE)
Poujois et al., 2013 [33]	19(8)	21(10)	63.8 ± 8.7	60.3 ± 8.0	NA	NA	NA	18.2 ± 9.6	35.3 ± 2.7	SPM2	VBA	*p* < 0.05 (corrected)
Cardenas-Blanco et al., 2016 [34]	34(12)	29(6)	58.0 ± 9.9	61.8 ± 10.0	NA	NA	NA	31.3 ± 21.3	35.1 ± 6.4	FSL	TBSS	*p* < 0.05 (FWE)
Crespi et al., 2014 [35]	19(6)	20(8)	60.4 ± 10.1	61.6 ± 7.5	NA	NA	NA	23.1 ± 20.6	39.9 ± 9.0	FSL	TBSS	*p* < 0.01 (FWE)
Kassubek et al., 2014 [37]	111(43)	74(33)	62.0 ± 11.0	59.0 ± 11.0	NA	NA	NA	16.0 ± 14.0	39.7 ± 7.4	TIFT	VBA	*p* < 0.05 (FDR)
Zhang et al., 2014 [38]	20(6)	21(6)	49.9 ± 9.4	48.6 ± 10.3	4+1^a^	15+1^a^	NA	19.4 ± 10.6	36.7 ± 4.9	FSL	TBSS	*p* < 0.05 (corrected)
b, Trojsi et al., 2015 [39]	18(9)	18(9)	59.2 ± 11.6	61.0 ± 8.1	2	16	NA	24.0 ± 19.2	36.3 ± 7.4	FSL	TBSS	*p* < 0.05 (corrected)
b, Trojsi et al., 2015 [39]	18(8)	18(9)	59.5 ± 10.6	61.0 ± 8.1	7	11	NA	25.2 ± 14.4	32.2 ± 8.6	FSL	TBSS	*p* < 0.001 (uncorrected)
b, Trojsi et al., 2015 [39]	18(7)	18(9)	62.4 ± 11.3	61.0 ± 8.1	4	14	NA	46.8 ± 25.2	31.6 ± 7.3	FSL	TBSS	*p* < 0.05 (corrected)

Note: NA, not available; ALSFRS-R, amyotrophic lateral sclerosis functional rating scale-revised; FWE, family-wise error; FDR, false discovery rate; SPM, statistical parametric mapping; FSL, functional MRI of the brain (FMRIB) software library; TIFT, tensor imaging and fiber tracking; TBSS, tract-based spatial statistics; VBA, voxel-based analysis.

a One patient had both bulbar and limb onsets.

b ALS patients were divided into 3 groups of clinical stage 2A, 2B and 3.

### 3.2 Regional differences in FA in all of the included studies

**Fig. 2:**
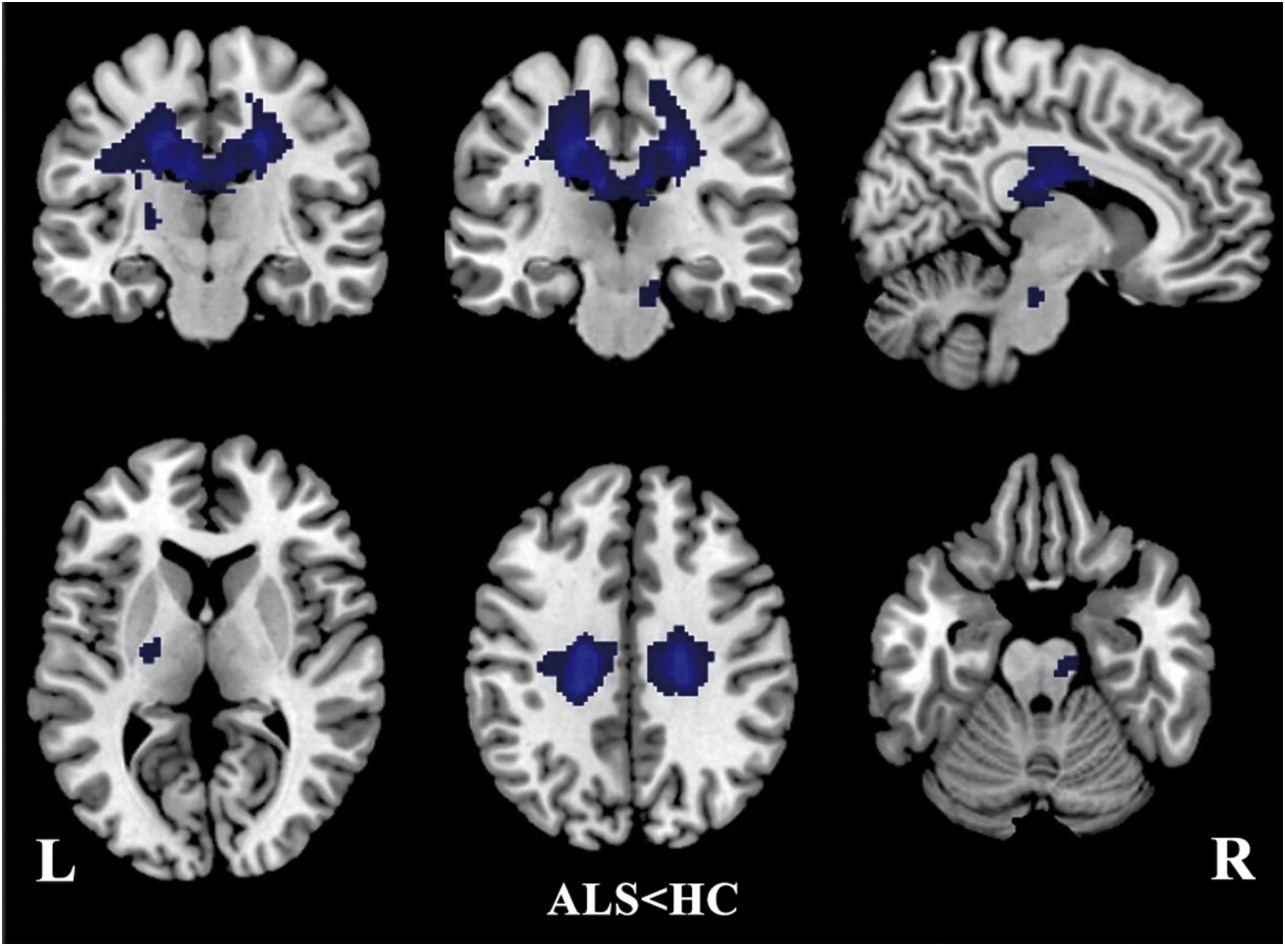
Regions showing FA reductions in the left corona radiata extending to the body and splenium of CC, left superior longitudinal fasciculus, posterior limb of internal capsule, right corona radiate, bilateral cingulate gyrus and the right corticospinal tract extending to right cerebral peduncle inpatients with ALS compared with healthy controls. Regions with decreased FA are displayed in blue. Significant clusters are overlaid on MRIcron template for Windows for display purposes only. CC = corpus callosum; FA = fractional anisotropy; ALS = amyotrophic lateral sclerosis. (For interpretation of the references to color in this figure legend, the reader is referred to the web version of this article.)

**Fig. 3:**
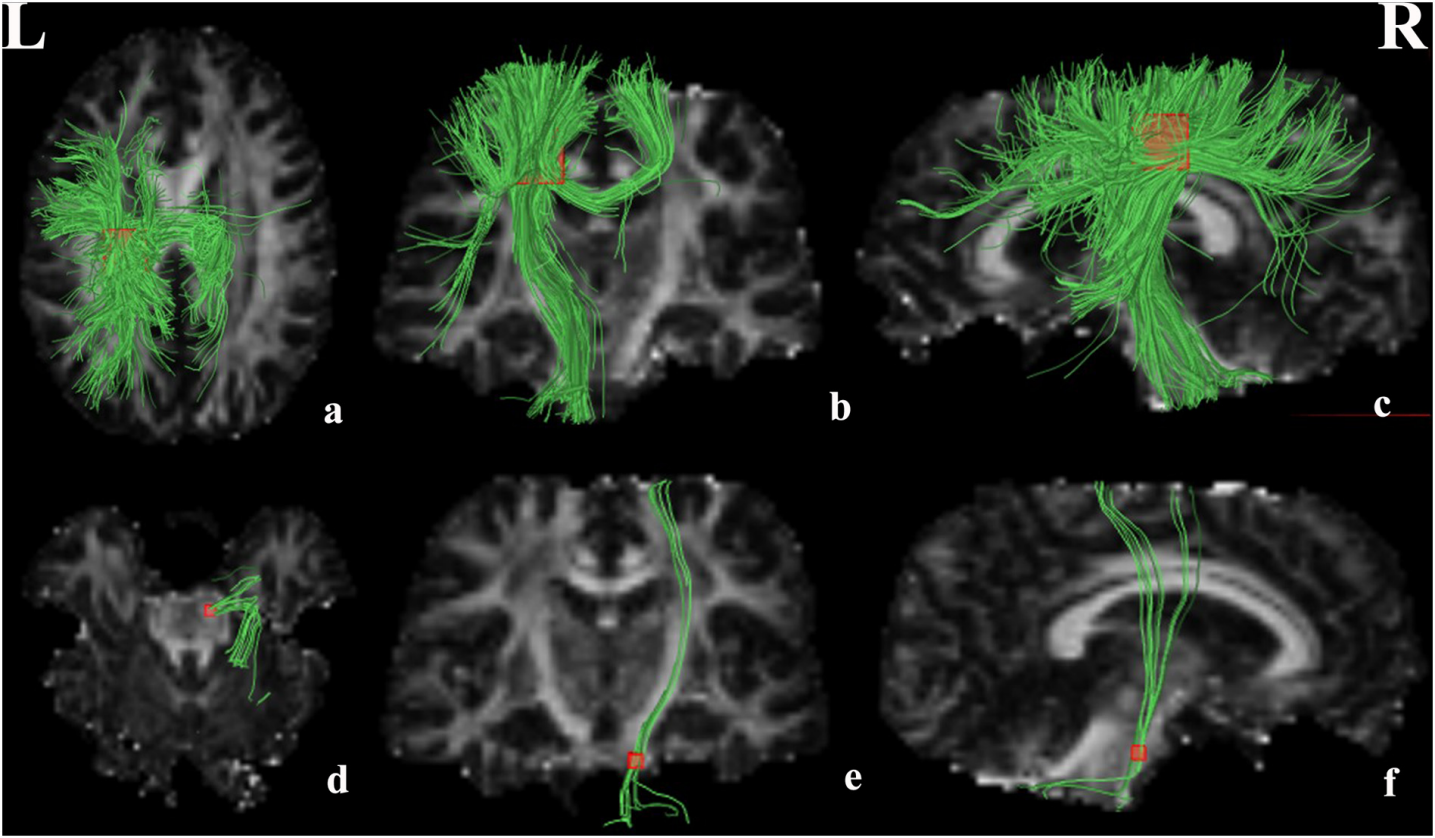
White matter tracts crossing these brain regions which showed decreased FA in patients with ALS. (a–c) Images showing the left corticospinal tract left superior longitudinal fasciculus and the interhemispheric fibers. (e–f) Images showing the right corticospinal tract. FA = fractional anisotropy; ALS = amyotrophic lateral sclerosis.

The pooled meta-analysis revealed that patients with ALS exhibited significant FA reductions in two clusters relative to healthy controls, as illustrated in Fig. 2 and Table 2. The largest cluster exhibited a peak coordinate in the left corona radiata that extended to the body and splenium of the CC, left superior longitudinal fasciculus, posterior limb of the internal capsule, right corona radiata, and bilateral cingulate gyrus. Fiber tracking indicated that the main WM tracts involved in this region were the left corticospinal tract, the left superior longitudinal fasciculus and the interhemispheric fibers running through the CC, which are represented as green tracts in Fig. 3a–c. The other cluster exhibited a decreased FA in the right corticospinal tract extending into the right cerebral peduncle, as illustrated by the green tracts in Fig. 3.

**Table 2:** Clusters of fractional anisotropy reductions in patients with amyotrophic lateral sclerosis relative to healthy controls.

Region	Maximum			Cluster		Jackknife sensitivity analysis
	MNI coordinates x, y, z	SDM value	*p*-Value	Number of voxels	Breakdown (number of voxels)	
Corona radiata_L	−18,−26,38	−4.471	~0	4030	Body and splenium of corpus callosum(878) Corona radiata_L(444) Corona radiata_R(366) Superior longitudinal fasciculus_L(202) Posterior limb of internal capsule_L(58) Cingulate gyrus L(54) Cingulate gyrus R(42)	16/16
Corticospinal tract_R	8,−22,−22	−1.839	0.003135739	50	Corticospinal tract_R(23) Cerebral peduncle_R(19)	12/16

SDM = signed differential mapping; MNI = Montreal Neurological Institute; L = left; R = right.

### 3.3 Reliability analysis

The whole-brain jack-knife sensitivity analysis of the pooled meta-analysis showed that the decreased FA in patients with ALS in the left corona radiata was highly replicable; this decrease was preserved throughout all 16 combinations of the datasets. The results regarding the right corticospinal tract remained significant in all but 4 combinations of the datasets (Table 2).

### 3.4 Subgroup meta-analyses

The subgroup analyses revealed that the cluster in the left corona radiata remained unchanged in all of the subgroup analyses. In the SPM (n = 9 datasets) and TBSS (n = 6 datasets) subgroups, the cluster in the right corticospinal tract remained unchanged, but the FSL/TIFT (n = 7 datasets) and VBA (n = 10 datasets) subgroup analyses failed to detect this cluster.

### 3.5 Meta-regression analysis

**Fig. 4:**
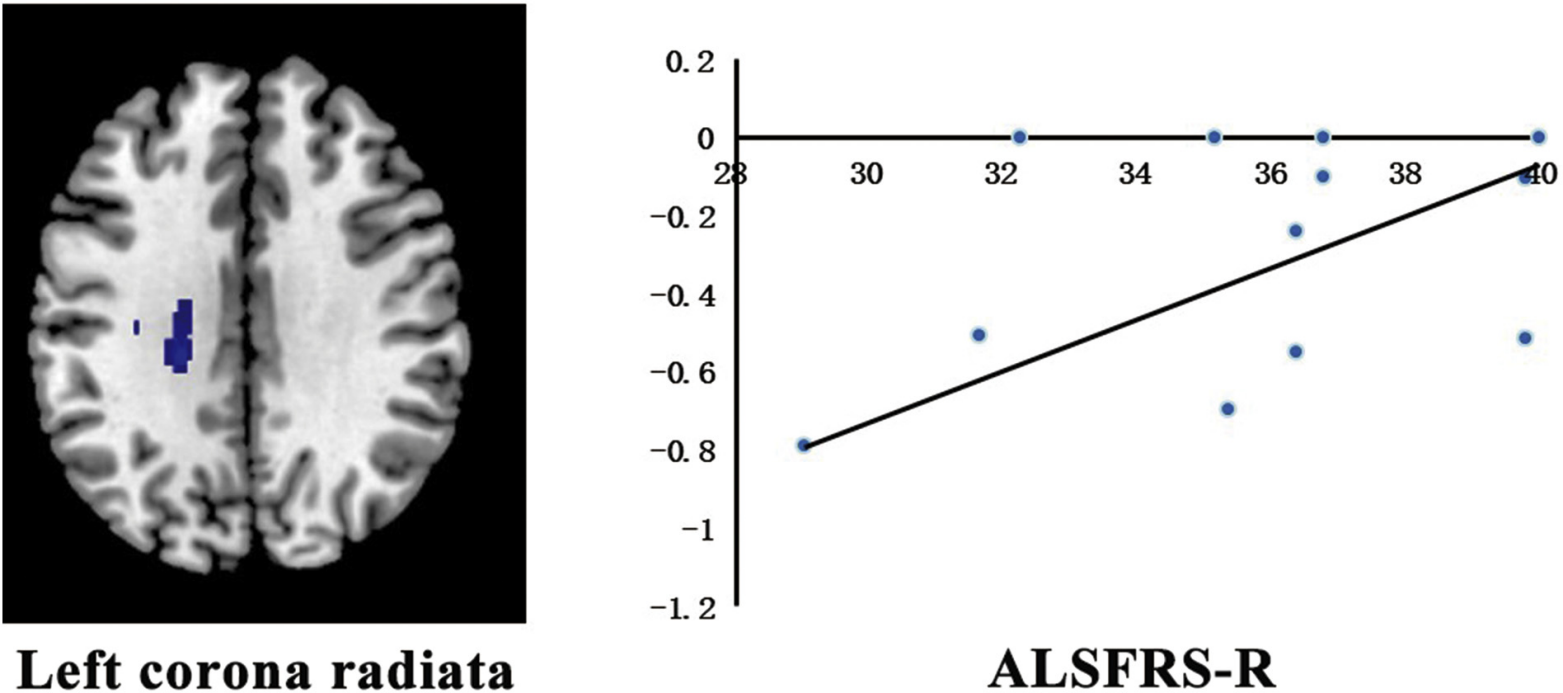
The result of the meta-regression analysis showing that the ALSFRS-R in patients with ALS is positively associated with decreased FA in the left corona radiate. FA = fractional anisotropy; ALS = amyotrophic lateral sclerosis; ALSFRS-R = amyotrophic lateral sclerosis functional rating scale–revised.

Meta-regression analysis found a positive correlation between the ALSFRS-R score and FA reduction in the left corona radiata (Montreal Neurological Institute coordinates: x = −22, y = −28, z = 30; AES-SDM value = − 3.188, *p* = 0.000023474; 349 voxels) as illustrated in Fig. 4. The mean age and illness duration were not linearly correlated with the FA reductions. The age at onset could not be investigated because none of the included studies reported these data.

## 4 Discussion

The pooled meta-analysis of the DTI studies revealed WM microarchitecture alterations indicated by FA decreased in patients with ALS compared to the healthy controls. This meta-analysis identified FA reductions in two clusters. The largest cluster exhibited a peak coordinate in the left corona radiata that extended to the body and splenium of the CC, left superior longitudinal fasciculus, posterior limb of the internal capsule, right corona radiata, and bilateral cingulate gyrus. The other cluster exhibited decreased FA in the right corticospinal tract that extended to right cerebral peduncle. Furthermore, the results of the reliability analysis and subgroup meta-analysis suggested the main results were largely unchanged. According to the meta-regression, the FA reduction in the left corona radiata reflected the severity of the disease to some degree.

In our meta-analysis, we found that the bilateral corticospinal tracts exhibited reduced FA. The corticospinal tract in the brain is the highlight of studies in patients with ALS. Regardless of whether ALS patients are examined at baseline or at follow-up, changes can always be found in the corticospinal tract. Regarding axial diffusivity, some studies have reported an increase (Metwalli et al., 2010), some groups have reported a decrease (Aoki et al., 2005), and some groups have reported a lack of changes (Agosta et al., 2010); thus, an increase is not consistently observed. One study found (Zhang et al., 2014) that the FA value of the left corticospinal tract was positively related to clinical function and able to predict disease progression. Because ALS patients exhibit FA reductions in the corticospinal tract regardless of the involvement of upper motor neurons, DTI may contribute to the early diagnosis of the disease (Sach et al., 2004). Decreased FA in the left corona radiata was found in this meta-analysis and indicates that the functionality of myelin and the fiber density have decreased. The ALSFRS-R score reflects the effects of upper and lower motor neuron disease on daily life activities. The ALSFRS-R score can be used to assess the patient's disease status in addition to predicting the patient's survival time. According to previous results, patients with higher scores have longer survival times (Zhong et al., 2011). In this meta-analysis, we found that FA reductions in the left corona radiata were positively associated with the ALSFRS-R score. Therefore, we suspect that the left corona radiata may be the key to survival. In the left corona radiata, there are many pathways that have been recognized as serving as junctions with the frontal cortex. The radial fibers extend from the internal capsule to the cerebral cortex. This anatomy may be related to the partial normalization of the conduction dysfunction on one side of the corona radiata, which means a signal not only activates the contralateral conduction pathway but also activates the ipsilateral conduction pathway (Zheng et al., 2008). FA reduction in the posterior half of the posterior limb of the internal capsule has also been reported (Sach et al., 2004) before. The decreased FA in the posterior limb of the internal capsule is in according with the pathological findings of the combined motor neuron disease and the localization of the corticospinal tract fibers in the internal capsule. Previous research also found a decrease in FA underneath the motor and premotor cortices in ALS patients (Sach et al., 2004). Furthermore, Davidoff (RA, 1990) found that lower FA in the motor cortex corresponds to changes in fibrous degeneration from the primary motor cortex; nearly 60% of pyramidal axons come from the primary motor cortex (Brodmann region 4), and the other pyramidal axons come from the anterior motor cortex (Brodmann region 6) and parietal lobe. Thus, the fibrous degeneration of the premotor cortex could lead to a decrease in FA below it, and this notion is consistent with the anatomical connections. Thus, the decreased FA in the posterior limb of the internal capsule may be related to the degeneration of the fibers emerging from the premotor cortex in addition to the demonstrated WM disintegration along the corticospinal tract.

Another intriguing finding of this meta-analysis is that an FA decrease was found in the interhemispheric fibers running through the CC. In the early stage of ALS, large Betz and smaller pyramidal neurons project their axons to form CSTs, which have an active and at least partly independent bilateral cortical degenerative process followed by secondary CC damage according to the corticosteroid model (Trojsi et al., 2015). According to previous research, the decreased FA in the CC reflects neuropathological findings of degenerated pyramidal tract bundles running across the middle body of the CC in patients with ALS (Sach et al., 2004). The CC connects the orbitofrontal and frontal cortices. Thus, this fiber damage might represent an additional mechanism that is responsible for the motor manifestations of the disease (Agosta et al., 2007) and may reflect cognitive impairments (Keil et al., 2012). Fiber changes in the CC have been reported to be related to negative emotions (Crespi et al., 2014). Other studies have reported that decreased FA and corresponding regional radial diffusivity increases within the major tracts of the CC are observed as a consistent feature of all four motor neuron disorders (Muller et al., 2012). Thus, FA changes in the CC may be an important indicator of robust regional damage.

According to our research, the left superior longitudinal fasciculus and bilateral cingulate gyrus exhibited FA reductions. ALS patients with dysphagia exhibit DTI abnormalities in these regions that reflect reduced cortical activation as well as the importance of planning the sequential movements of swallowing (Li et al., 2009). Previous studies have demonstrated significantly decreased FA in the bilateral cingulate gyrus. One study (Smith, 1960) found a significant FA reduction in the bilateral cingulate gyrus and neurodegeneration in the cingulate. FA reductions in the right posterior cingulate gyrus (BA31) and left anterior cingulate gyrus (BA32) (Lule et al., 2010) have been reported. These findings are inconsistent with our results, and the discrepancies may be attributable to the sample size and degree of the disease. According to this research, the cingulate gyrus is connected to multisensory areas, and these connections may occur in the primary sensory cortex and parietal lobe between the primary receptors that are experiencing secondary degeneration. In the advanced stages of ALS, compensatory activities in the reported association areas may be affected at this functional level of the denervation (Lule et al., 2010). The findings reveal that the severity of bilateral upper motor neuron impairment is correlated with perfusion in the midcingulate cortex in ALS patients (Rule et al., 2010). Both the cingulate gyrus and the superior longitudinal fasciculus play crucial roles in emotion processing, initiation, motivation, and goal-directed behaviors (Devinsky et al., 1995). Thus, impairments of these two structures contribute to emotion liability and cognitive disorders in patients with ALS (Maddock et al., 2003).

We found FA reductions in the right cerebral peduncle, which is consistent with a previous study (Poujois et al., 2013). A diffusion tensor tractography study showed that the CSTs traversed the mid to lateral portions of the cerebral peduncle (Park et al., 2008). The reduction of FA in the right cerebral peduncle may have a relationship with the FA decrease in the right CST. The WM in the cerebral peduncle is a major pathway through which the cerebral cortex manages spontaneous movements and exerts its effects on the cerebellum. One study found that the burden of upper limb movement defects is greater on the right side, which agrees with the FA reduction observed in the left CST of ALS patients compared with controls, and that 34.2% of patients showed a reduction in the right cerebral peduncles (Poujois et al., 2013).

## 5 Limitation

There are still some limitations in the current study. First, similar to previous voxel-based meta-analyses, small sample size limits the generalization of results, especially in subgroup analyses. **Second, the included studies differed in MR scanner venders, field strength, scanning parameters. It is impossible to eliminate these differences by statistical means**. Third, there was no assessment of the relationship between age at onset and ALS, and more data on age at onset are needed. **FA combined with other diffusion parameters (MD, AD, and RD) is more helpful for illuminating alterations in white matter microstructure. Unfortunately, most of the studies included in the present meta-analysis did not report the alterations in other diffusion parameters**. Finally, another limitation is related to document bias. Although we attempted to include as many studies as possible (even if they were negative), the possibility of publication bias could not be ruled out altogether.

## 6 Conclusion

In summary, this meta-analysis revealed two consistent clusters of FA reductions in patients with ALS. The largest cluster was located in the left corona radiata, extending to the body and splenium of the corpus callosum, left superior longitudinal fasciculus, posterior limb of the internal capsule, right corona radiata, and bilateral cingulate gyrus, as well as involving the left corticospinal tract, left superior longitudinal fasciculus and the interhemispheric fibers running through the CC. The other cluster of decreased FA was in the right corticospinal tract, extending to the right cerebral peduncle. Furthermore, the FA reduction in the left corona radiata can reflect the severity of the disease to some degree. This study provides a thorough profile of WM microarchitecture alterations in ALS and further evidence that the neuronal degeneration is not limited to the corticospinal tract but also includes extra-motor areas, which supports the view that ALS is a multisystem degenerative disorder involving the WM.

## Conflict of interest

The authors declare no conflict of interest.

## Appendix A Supplementary data

Supplementary material Image 1
