# Supplementary material for: Altered white matter microarchitecture in amyotrophic lateral sclerosis: A voxel-based meta-analysis of diffusion tensor imaging
Source: Neuroimage Clin. 2018 Apr 4;19:122–9. doi: 10.1016/j.nicl.2018.04.005 (PMC6051469; doi:10.1016/j.nicl.2018.04.005)
Supplement: Supplementary file 1 — Supplementary material [file mmc1.docx]

Abe.spm_mni

32,-16,26,-9.15

-34,-10,28,-7.47

Agosta.spm_mni

14,-12,-10,-3.47

-12,-20,-14,-4.49

20,-20,26,-4.59

-18,-22,28,-3.61

Cardenas.spm_mni

None

Crespi.spm_mni

20,-20,49,n

21,-20,41,n

Kassubek.spm_mni

-16,-20,31,n

16,-20,31,n

Keil.spm_mni

-10,-14,29,-6.03

-1,-16,25,-5.35

11,-16,29,-5.29

-8,-27,-34,-5.82

9,-26,-36,-4.54

10,-22,-24,-3.88

14,-21,58,-5.69

27,-38,50,-4.02

-12,-13,70,-5.03

48,4,8,-4.59

-35,-12,48,-4.37

-39,-19,48,-4.18

-10,-37,61,-4.35

-42,-15,23,-4.34

-28,-24,55,-4.26

-17,-24,55,-3.62

33,-8,38,-3.87

-39,-6,35,-3.86

-46,1,40,-3.82

-20,-48,-11,-3.80

Poujois.spm_mni

-21,-21,39,-5.37

Sach.spm_mni

21,15,6,n

24,12,12,n

1,12,24,n

9,12,4,n

24,18,39,n

21,21,42,n

18,18,60,n

21,3,42,n

42,3,33,n

9,18,21,n

6,15,21,n

Sage.spm_mni

-12,-26,58,-4.98

12,-26,62,-3.09

-42,-14,50,-4.73

32,-16,48,-3.55

-20,-22,44,-3.39

24,-22,46,-3.51

22,-14,6,-3.55

16,18,-16,-3.96

-16,26,-16,-3.24

-16,14,44,-3.23

38,24,28,-3.23

10,56,-4,-3.16

22,32,-2,-3.16

-32,-14,-14,-3.02

34,-10,-16,-4.36

44,12,8,-3.45

-48,6,10,-3.31

-10,-52,40,-5.15

-52,-34,30,-3.46

48,-54,34,-3.42

-8,-6,56,-3.94

-34,10,40,-3.37

Senda.spm_mni

36,-26,26,-6.10

-14,-20,46,-4.73

Thivard.spm_mni

40,-24,44,-5.35

36,-14,44,-4.11

38,-8,36,-3.87

-30,-20,42,-5.55

-18,-22,30,-5.98

-24,-22,48,-5.41

20,-22,42,-3.94

-18,-20,-6,-4.75

14,-22,52,-4.69

0,-18,12,-4.54

42,-44,28,-5.01

-44,-50,28,-3.70

-42,-2,2,-4.40

-42,-8,18,-3.85

40,10,-2,-3.68

Trojsi_2A.spm_mni

0,-9,28,-2.58

-22,-33,35,-2.32

-39,-14,30,-2.49

Trojsi_2B.spm_mni

-39,-14,30,-1.68

-28,-72,-2,-1.92

-23,-8,10,-2.54

Trojsi_3.spm_mni

0,-9,28,-2.18

-7,-23,-29,-2.10

-22,-33,35,-2.15

2,-28,36,-1.70

-8,-9,3,-1.77

7,-9,4,-1.90

-39,-14,30,-2.05

50,-5,27,-1.99

-30,-69,-3,-1.92

31,-69,-3,-1.89

-28,-72,-2,-1.91

29,38,3,-1.79

27,13,-7,-1.68

-23,-8,10,-1.81

26,-7,-9,-2.25

Zhang.spm_mni

-28,-17,24,-3.87

25,-16,35,-3.49

12,-20,-21,-3.49

-21,-15,5,-3.06

43,-3,28,-2.64

-9,-14,28,-2.43

14,-22,30,-1.80

Zhang_hui.spm_mni

57,80,37,-1.77

76,52,40,-2.37
